# Coastal Land Use Management Methodologies under Pressure from Climate Change and Population Growth

**DOI:** 10.1007/s00267-022-01705-9

**Published:** 2022-08-27

**Authors:** Tao Wu, Juliana Barrett

**Affiliations:** 1 Department of Landscape Architecture, Nanjing Agricultural University, 1 Weigang, Nanjing, Jiangsu 210095 China; 2 Department of Plant Science and Landscape Architecture, University of Connecticut, 1376 Storrs Road, Storrs, CT 06269 USA; 3 Connecticut Sea Grant, 1080 Shennecossett Road, Groton, CT 06340 USA; 4 Department of Extension, College of Agriculture, Health and Natural Resources, University of Connecticut, 1376 Storrs Road, Storrs, CT 06269 USA

**Keywords:** Land use change, Climate change, Resilience, Adaptive management, Social-ecological system

## Abstract

Throughout history, humans living in the coastal area constantly adapt to the natural environment and create a changing environment. The rapid coastal development occurred in the mid-19th century and peaks in the mid-20th century, which was a common process in most industrialized areas. With increasing population growth and urban sprawl, many coastal lowlands are unprecedently vulnerable to climate change impacts such as sea level rise, increasing extreme storm events, and coastal flooding. Under the influence of urban revitalization and conservation, the landward shoreline movement accelerated and coastal land shrank, accompanied by community retreat. This research focuses on the importance of incorporating an understanding of the changing coastal land-ocean interaction into adaptive management strategies by illustrating the relationship of land use change, social-economic development, and climate change. Typical coastal changes in Connecticut were selected: New Haven Harbor reflects a dramatic seaward land accretion under industrial and transportation development, New London downtown waterfront reveals a trend of building retreat under industrial and commercial transformation and coastal hazard, New London Ocean Beach indicates how overdeveloped coastal low-lying community fully retreat after a natural disaster, and Jordan Cove barrier island shows a highly dynamic coastal land change and proactive management strategy. The results reveal that to cope with a constantly changing shoreline and the challenges of climate change, a resilient management process must incorporate a cycle of learning, experimenting, and creating with the goal of developing new solutions that are able to deal with our ever-changing environment.

## Introduction

The coastal zone is home to both intensive human activity and fragile ecological areas. Coastal regions and populations are exposed to pressures and hazards from both land and ocean, making the coastal zone the most transformed and imperiled social-ecological system on the earth (Ramesh et al. 2015). Coastal zones are not only exposed to storms, land subsidence, sea level rise (SLR), saltwater intrusion, and other environmental risks, but are also under pressure from rapid urban and industrial expansion, population growth, and over-exploitation of natural resources (Xu et al. 2016). Land-Ocean Interactions in the Coastal Zone (LOICZ), established by the International Geosphere - Biosphere Programme (IGBP), provides science knowledge about changes in land use, sea level and climate, and the impacts on coastal. The new vision of LOICZ is to support transformation to a sustainable and resilient future for society and nature on the coast, and to help coastal zone managers and local governments make better decisions.

Shorelines continuously migrate in response to winds, waves, tides, sediment supply, changes in relative sea level, and human activities. As a result, shoreline changes are generally not constant through time and frequently switch between erosion (landward migration) and accretion (waterward migration) and vice versa. Cyclic and non-cyclic processes change the position of the shoreline over a variety of timescales from the daily and seasonal effects of winds and waves to decadal or longer changes in sea level (O’Brien et al. 2014). As a vital geological agent, anthropogenic activity has greatly influenced the coastal zone, including the filling of coastal wetlands, conversion of coastal habitats, and coastal hard structures such as seawalls, groins, and bulkheads. Thus, future social and economic coastal development remains threatened by a variety of factors, both natural and human (Dinan 2017, Brattland et al. 2019, Fan et al. 2006).

Urban sprawl, with a continuous increase in built-up areas, can result in the loss of natural lands and wildlife (Chunwate et al. 2019a, b) and can increase the risk of flooding due to the essential elements of exposure, vulnerability, and hazard. Similarly, higher population growth can lead to increased impervious surfaces and higher runoff(Azzam and Belhaj Ali 2019; Mustafa et al. 2018). The significant changes in physical dangers are accompanied by urbanization patterns and population and development pressures that are placing even more people and property in harm’s way (Beatley 2009). In cities and urbanized areas, fragmentation of natural lands, disruption of hydrological systems, and alteration of energy flow and nutrient cycling reduce the resilience of urban ecosystems, and leave systems increasingly vulnerable to shifts in system control and structure (Alberti and Marzluff 2004).

This research asks questions about 1. what can people learn from the intrinsic nature of highly dynamic coastal landscapes and the intensification of coastal change caused by climate change? 2. why is it important to incorporate an understanding of the changing coastal land-ocean interaction into urban planning and adaptive management, especially when dealing with large-scale social, environmental, and economic problems of our still young and rapidly evolving coastal landscape? This research seeks to discern how coastal land use changes under coupled forces from both human and nature, and what is the trend of these changes. Through a spatial and temporal CLUC analysis, it seeks to provide a basis and reference for future coastal land use management and decision making with a resilient and sustainable approach.

The visions of sustainable development and resilient city provide theories for coastal management. Sustainability is the capacity to create, test, and maintain adaptive capability. Development is the process of creating, testing, and maintaining opportunity. The phrase that combines the two, “sustainable development,” thus refers to the goal of fostering adaptive capabilities and creating opportunities (Holling 2001; Gunderson and Holling 2002). Adaptive management and sustainable development need resilient thinking on coastal management (Sellberg et al. (2018)), which can be found at all levels of decision-making in Rotterdam, Netherland (Lu and Stead 2013). Resilient thinking provides a new way of framing and responding to uncertainty and vulnerability in coastal spatial planning and urban development. It offers an alternative paradigm for developing strategies and approaches to deal with large-scale social, environmental, or economic change in cities; in the Dutch context, this is represented by a paradigm shift from “keeping feet dry” to “living with water” (Lu and Stead 2013).

Resilience, in both its social and ecological manifestations, is an important aspect of the sustainability of development and resource utilization. A simple definition of resilience is the ability of a city to absorb disturbance while maintaining its functions and structures (Holling 1987, 2001). The notion of resilience is often associated with the ability to learn, in order to become more robust to change (Newman (2009)). Currently, the issues of resilience and vulnerability are becoming more important in the framing of resource management questions. In policy terms, these are useful since both ecological stability and resilience are perceived as desirable social goals for many issues, from nature conservation to climate change (Adger 2000; Ahern 2011). Nature-based solutions (NBS) are increasingly being applied to guide the design of resilient landscapes and cities to enable them to reach economic development goals with both beneficial outcomes for the environment and society (Lafortezza et al. 2018). Natural infrastructure often provides similar services as well as added benefits that support short- and long-term biological, cultural, social, and economic goals (Powell et al. 2018).

## Methods

**Fig. 1:**
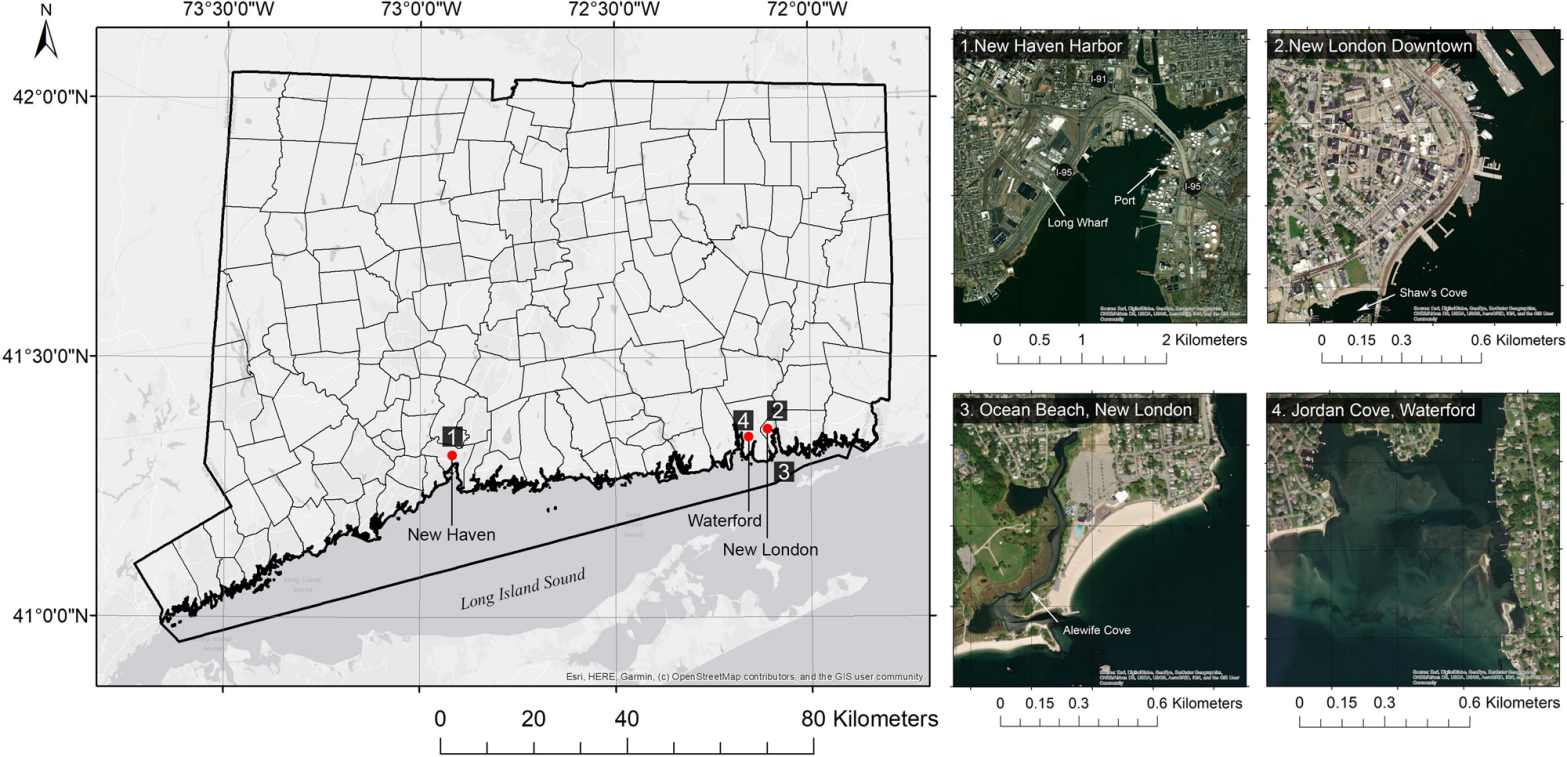
Location of the research sites. Left: Four research sites in the state of Connecticut. Right: (1) New Haven harbor. (2) New London downtown waterfront. (3) Ocean Beach of New London. (4) Jordan Cove of Waterford

Connecticut has a coastline of 96 mi (155 km) with 24 towns along the Long Island Sound (LIS) shoreline, including several of the largest and most urban in the state. Most of the coastal towns have large areas that are less than 20 feet (6 m) above sea level. Based on climate modeling and analogies with glaciological conditions, the global mean SLR projections by 2100 range between 0.8 (2.6 feet) and 2.0 m (6.5 feet) (Pfeffer et al. 2008). However, the rates of SLR observed in Long Island Sound, Connecticut, are more than 50% higher than the global average during the same time period, which is about 1.6 m (6.5 inches) per century. Connecticut SLR projection of NOAA report (O’Donnell 2019) anticipates a sea level rise of 0.5 m (1 ft 8 inches) in Long Island Sound by 2050. Under the pressure and disturbance of coupled natural forces and human activities, these coastal towns experienced significant changes in land use and morphology over the past several centuries. Four sites along the Connecticut coast representing typical land use change were used in this study to examine and analyze CLUC (Fig. 1):

- New Haven Harbor (41° 17’ 34” N, 72° 55’ 22” W): seaward land accretion under dramatic industrial and transportation development, then retreat due to sea level rise and flooding
- New London downtown waterfront (41°21’ 15” N, -72° 05'33” W): seaward land accretion with a trend of building retreat under industrial and commercial transformation and coastal hazard
- New London Ocean Beach (41° 18’ 28” N, 72° 5’ 56” W): overdeveloped coastal low-lying community and full retreat
- Jordan Cove (41° 18’ 46” N, 72° 9’ 3” W) in Waterford: highly dynamic barrier island change and proactive coastal planning and management strategy

The historic maps from the 1840s to1920s were incorporated in this study to show the temporal scale of land use change for the four sites. The typical time series of land change in historic map study include 1840–1850s, 1890s, and 1910–1920s. The data sources are from the United States Geological Survey (USGS), the University of Connecticut’s Map and Geographic Information Center (UConn MAGIC), and the Connecticut State Library archives. The aerial images of 1934, 1951, 1970, 1986, and 1995 adopted in this study are provided by Connecticut State Library. The aerial photos of 2004, 2006, 2008, 2010 are accessed via UConn MAGIC. The aerial imagery of 2012, 2014, 2016 are from National Agriculture Imagery Program (NAIP) of the U.S. Department of Agriculture (USDA). The most recent aerial imagery of 2019 is accessed via Environmental Systems Research Institute (ESRI). Social and economic data including population, urban planning and development is retrieved from journals, reports, books, and local news and they are referenced appropriately. The GIS data of Digital Elevation Models (DEM) is incorporated for the vulnerability analysis, which is acquired from Connecticut Environmental Condition Online (CT ECO). Archival data are included to gain a well-rounded background understanding of an area (Connecticut State Library 2013).

The georeferencing of historic maps and satellite images in GIS is a critical measure for comparison and analysis of coastal land geomorphic change and the quantitation of these changes. Most of the historic maps and early air photos are in picture format without spatial reference or use varied coordinate systems, while this study applied georeferencing techniques to assign real-world coordinates to each pixel of these raster images making the time series maps accurate and comparable. The georeferenced maps use projected coordinate system NAD 1983 (2011) StatePlane Connecticut FIPS 0600.

## Results

### New Haven Harbor: Seaward Land Accretion under Dramatic Industrial and Transportation Development and Retreat due to Sea Level Rise and Flooding

Most of the New Haven Harbor area is low-lying land less than 3 m in elevation and the land use is historically industrial. Before human interventions, these areas were largely open water. Beginning in the 1850s, the city of New Haven expanded as the city’s manufacturing industries began to flourish and experienced its greatest growth in population between 1890 and 1920. Under the stimulation of the Housing Acts of 1949 and 1954 and Highway Act of 1956, which triggered urban renewal programs throughout the United States, New Haven began to focus on transportation issues and the construction of the interstate highway system including I-91 and I-95. The former open water was largely filled to construct interstate highway around 1950s. The filled coast land was rapidly developed as an industrial area and transportation hub. From 1846 until the 1970s the shoreline has moved seaward by an average of 710 m with a total land-filled area of 206.7 ha. The dramatic land use change in New Haven harbor is highly correlated to the development of transportation and industry.

**Fig. 2:**
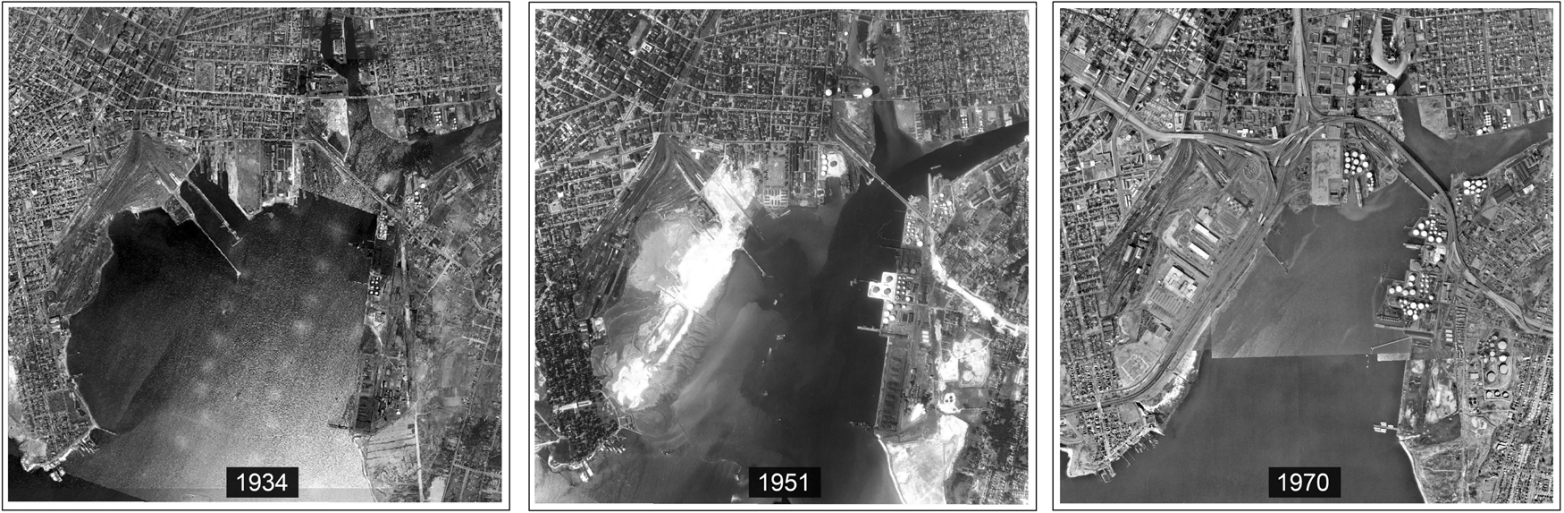
New Haven harbor area changes from 1840s to 2010s. Left: New Haven harbor in 1934. Middle: Long Wharf under construction in 1951. Right: Long Wharf industrial area and the interstate transportation system formed under dramatic coastal landfills

From the 1960s through the late 1990s, the central areas of New Haven continued to decline both economically and in terms of population, while suburban and coastal areas converted to more developed with the convenience of transportation and industrial development. As manufacturing has dropped after its heyday in the 1980’s, former manufacturing buildings in the harbor area have increasingly become large-scale retail, educational, and public use. The revitalization and regeneration of New Haven in the 1990s-2000s brought vitality back to the city, with an added focus on sustainable development and smart growth. The city’s 2002 Harbor Plan emphasized a balance of economic development, environmental sustainability, and cultural enrichment along the waterfront. Now New Haven harbor is designated as a mixed-use community with new green infrastructure incorporated in the framework of a coastal resilience plan due to flood issues along the waterfront (City of New Haven 2017) (Fig. 2).

**Fig. 3:**
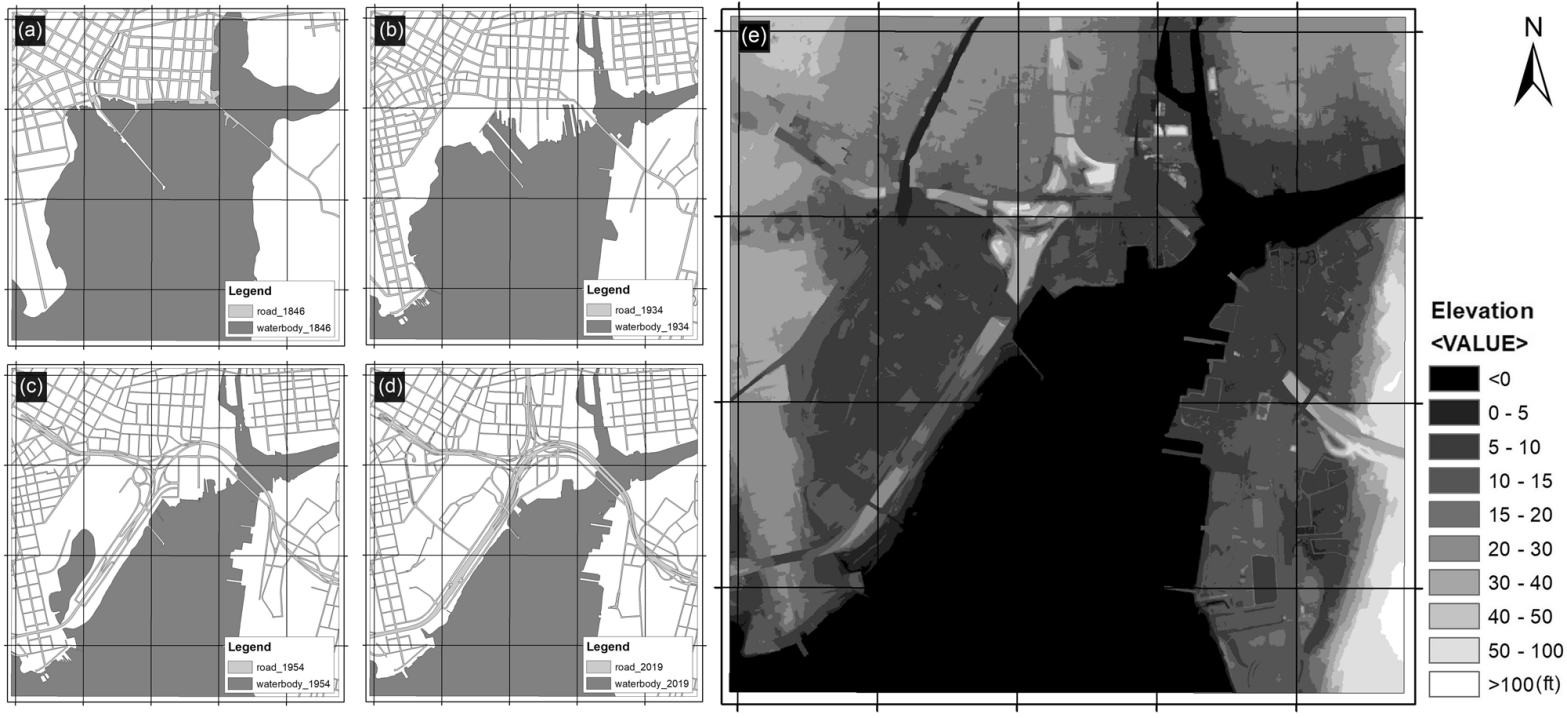
The time series of New Haven harbor changes from 1846 to 2019 and the current elevation of the harbor area. **a** 1846, **b** 1934, **c** 1954, **d** 2019, **e** Digital Elevation Model (DEM) of the New Haven harbor

In recent decades, affected by accelerated SLR, the shoreline has shown signs of landward movement. However, land loss and conservation along the waterfront area are interwoven after the 1990s under human interventions to protect lands from flooding and further loss. According to the City of New Haven City Plan Department (2017) report, the areas that experienced repeated flooding in New Haven are mainly located in low-lying artificially filled areas along the shoreline, most of which are industrial land use with a number of vacant lots. Nearly 60% of the artificial-filled land (191 ha, elevation < 10 ft (3.0 m)) in the harbor area is in the 100-year flood zone, and 80% of the artificial-filled land (256 ha, elevation < 12 ft (3.6 m)) is in the 500-year flood zone (Fig. 3). As sea level rises, storm surges from hurricanes and nor’easters are predicted to reach further inland as they are starting from a higher base. By the end of this century, it is possible that a Category 1 storm surge will be similar to what is now a Category 3 storm surge and a 500-year flood would happen on the frequency of a 100-year flood. Facing the changed environment and coastal hazards, the city of New Haven initiated Long Wharf Responsible Growth Plan as a smart growth path and the coastal resilience plan as a proactive adaptation policy (Perkins Eastman 2022).

### New London Downtown Waterfront: Seaward Land Accretion with Buildings Retreat under Industrial and Commercial Transformation and Coastal Hazard

**Fig. 4:**
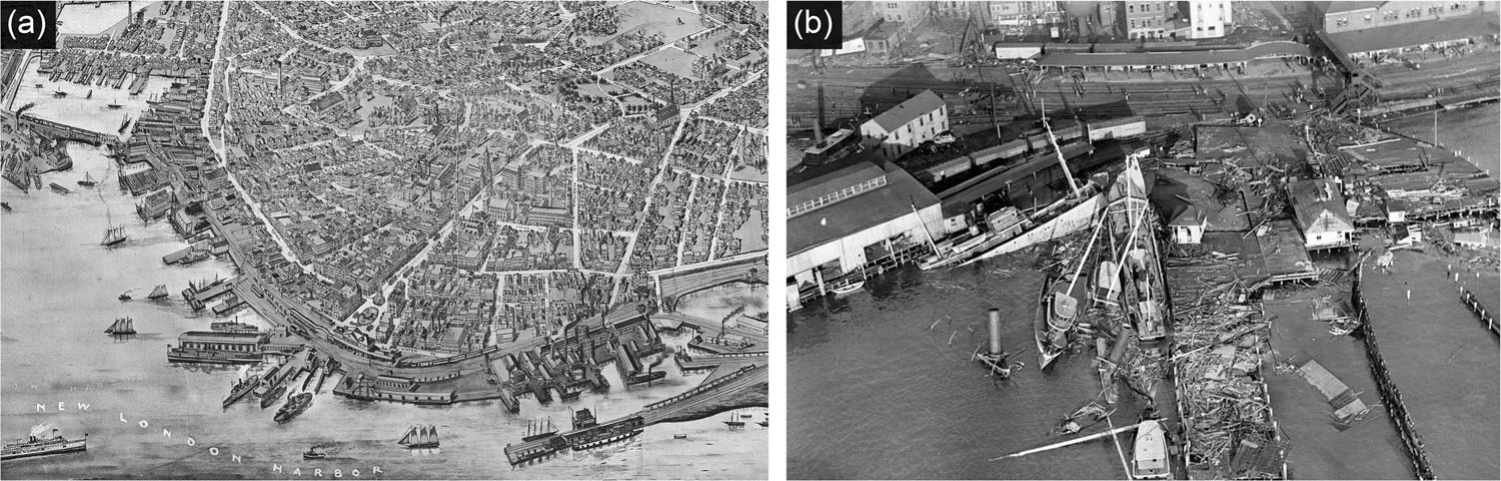
The 1938 New England hurricane destroyed most of the coastal buildings and infrastructures. **a** In the historic perspective map of 1911, a large number of buildings lined the shoreline with high-rise structures (from the Library of Congress). **b** In the devastating 1938 New England hurricane, most buildings and infrastructures along the shoreline were destroyed by wind, floods, and fires (from Connecticut State Library)

New London downtown waterfront is a seaport on the northeast coast of the United States and iconic for its long history of whaling and as a shipyard. The historically industry-based city’s social-economic system decisively influenced the downtown waterfront land use and transformed it as the economic concerns changed. Land use of the downtown waterfront experienced low density of residential to high density of industrial land uses from the 1850s to 1940s. The 1938 New England hurricane wreaked havoc on New London, most of the coastal structures were destroyed, and buildings directly on the shoreline vanished (Fig. 4). Since the 1950s, impacted by the industry transformation and extreme weather events, the downtown waterfront industrial land use decreased while residential and public use increased. Facing the problem of coastal industry decline in the 1960–1970s, the city created several urban renewal plans which focused on clearing deteriorating areas to create developable land for new constructions. Under this clearing and transformation action, the footprint of buildings sharply declined, recreational and commercial land use increased.

In the early 1980s, the urban management focused upon revitalizing the city’s downtown area, specifically on the coastal area and city parks. From the beginning of 21st century, due to more frequent coastal flood events hitting the coastal area and with impacts intensified by SLR (City of New London 2017), the waterfront community began to retreat and gradually transformed into low density mixed-use district with critical infrastructure and coastal open space. In the early 2000s, New London took advantage of smart growth development trends to continue revitalization and conservation efforts, the city’s waterfront area regained its vitality with mixed-use and public activities (New London Planning and Zoning Committee 2017). The downtown waterfront becomes a densely developed area and has substantial commercial and residential development with many historic resources along the shoreline now. This area faces the continual challenge of flooding, with both residents and municipal officials considering various forms of retreat, such as limiting new development in the flood area, allowing vacant lots along the shoreline to be constructed as green space. New London planning and zoning regulations seek to limit additional development in hazardous locations along the waterfront. Hazard mitigation and adaptation measures include land acquisition and open space preservation; and acquisition of floodplain is encouraged as a municipal priority (City of New London 2005).

**Fig. 5:**
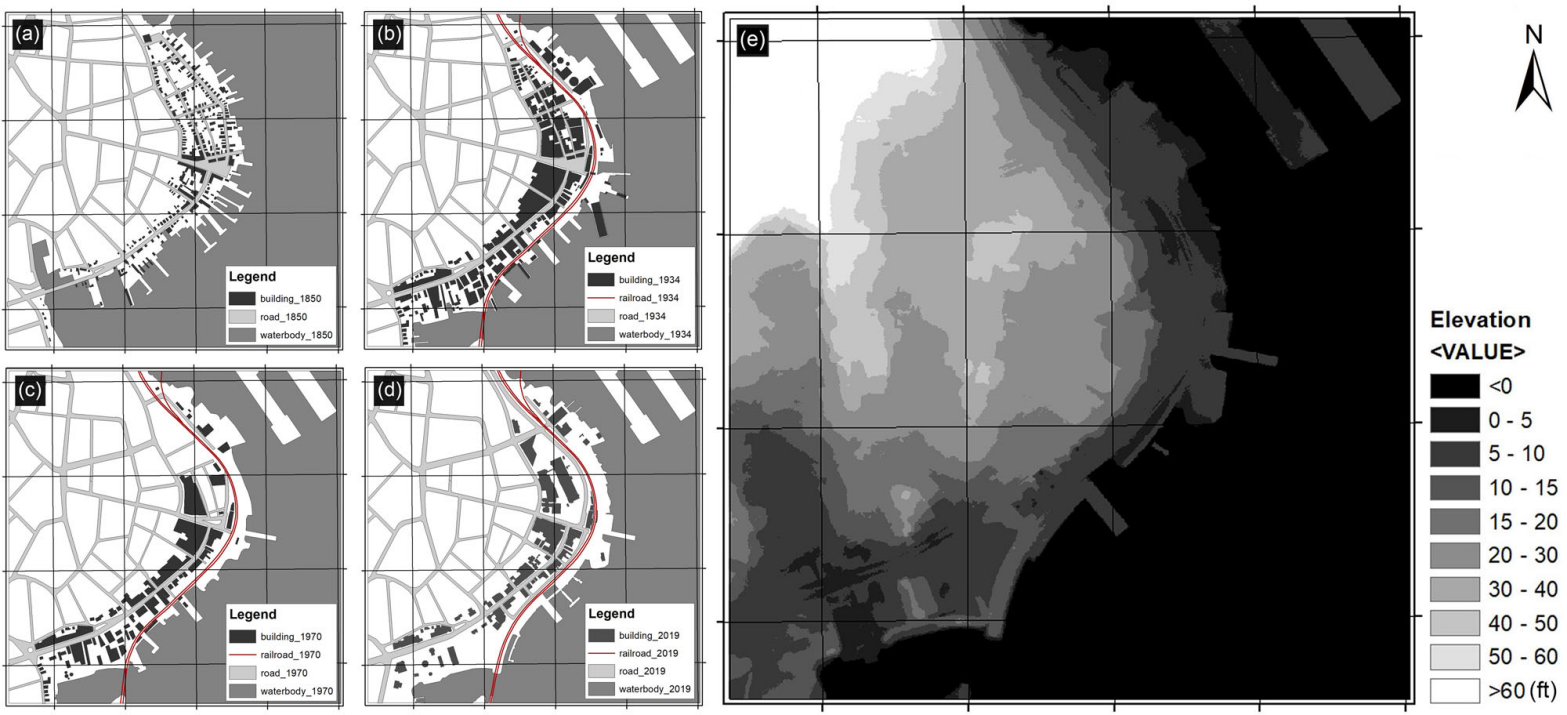
The time series of New London downtown waterfront changes from 1850 to 2019 and the current elevation of the downtown waterfront area. **a** 1850. **b** 1934. **c** 1970. **d** 2019. **e** Digital Elevation Model (DEM) of the New London downtown waterfront

The footprint of buildings along the downtown shoreline dropped from 90,564 m^2^ in 1934 to 56,650 m^2^ in 1970, and 52,997 m^2^ in 2019 (Fig. 5). The change not only indicates the reduction and decentralization of the buildings, but also functional retreat. Some properties in the lowest elevations had buildings partially demolished or abandoned the function of the first floor due to repeated flooding. To mitigate tidal flooding and hurricane surge hazards, the United States Army Corps of Engineers constructed a hurricane barrier that was completed in 1986 (U.S. Army Corps of Engineers USACE 2013; Morang 2016). As a result of the rising sea and uncertainty of extreme weather events deemed to exacerbate the already serious impacts on coastal properties, the artificially filled land behind the hurricane barrier is projected to be in the 100-year flood zone by 2050. As a pilot project of Resilient Connecticut, New London waterfront vulnerability assessment and resilience planning were implemented in 2018 by Connecticut Institute for Resilience and Climate Adaptation. The coastal management benefited from this project and incorporated the research products into future development and climate adaptation.

### New London Ocean Beach: Overdeveloped Coastal Low-lying Community and Full Retreat

**Fig. 6:**
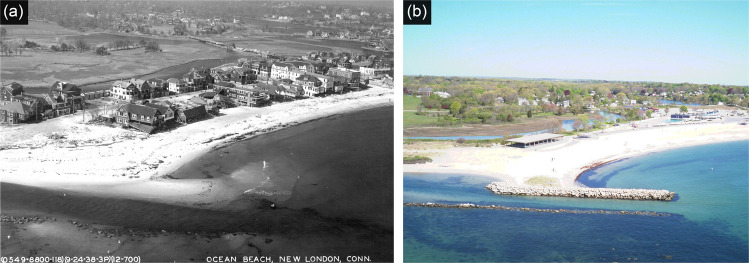
New London Ocean Beach community relocated after the 1938 New England hurricane. **a** Damage from the 1938 hurricane at Ocean Beach (from Connecticut State Library). **b** Ocean Beach Park in 2012 (photo credit Joel Stocker)

Located on a sand spit along the LIS and adjacent to a coastal lagoon, New London Ocean Beach area experienced rapid development followed by fully managed retreat through eminent domain (Fig. 6). Development of Ocean Beach began in the 1880s, accelerated in the early 20th century and peaked in the 1930s with more than 200 structures crowded on the beach (Zavar and Hagelman 2018). After the catastrophic 1938 New England hurricane destroyed most of the structures, the city acquired the land and transformed it into a park for public use. The city viewed the hurricane as an opportunity to remove the nuisances and restore the area to the former public beach. Despite objections from the owners of the Ocean Beach properties, the acquisition of property to reduce public vulnerabilities was widely supported and viewed as progressive. Through this acquisition, New London reduced vulnerability to hurricanes and storm surges in the Ocean Beach area (Zavar and Hagelman 2018).

**Fig. 7:**
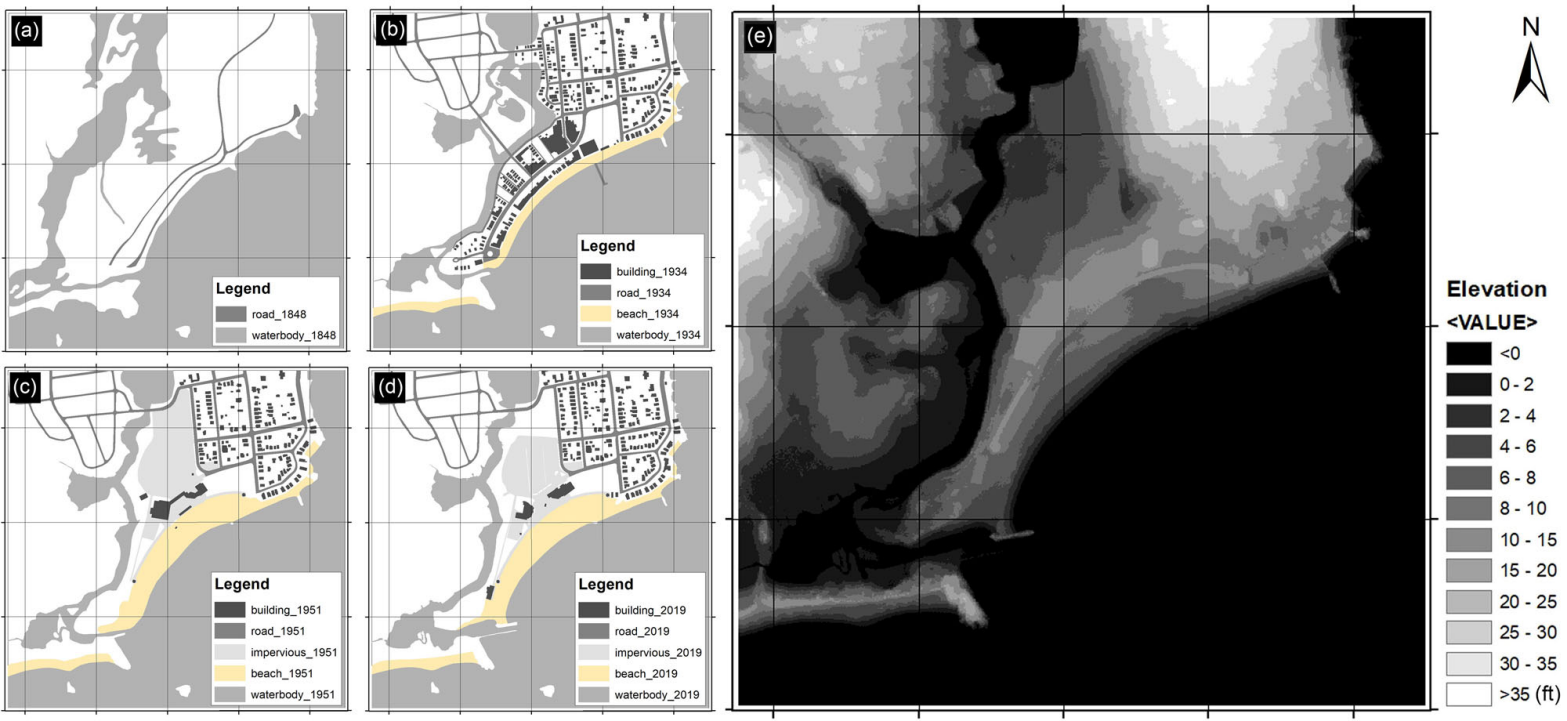
The time series of Ocean Beach changes from 1848 to 2019 and the current elevation of the Ocean Beach area. (**a**) 1848. (**b**) 1934. (**c**) 1951. (**d**) 2019. (**e**) Digital Elevation Model (DEM) of the Ocean Beach area

Other than the building reduction on the beachfront, the coastal lagoon and tidal marsh also changed dramatically in morphology and area (Fig. 7). The new Ocean Beach Park as green infrastructure opened in 1940 with the ability to absorb storm surges and floodwaters to some extent, maintaining the value of public entertainment and the resiliency for unpredictable coastal hazards. While construction of the parking lot significantly compromised the resilience of the wetlands and ecological function, transforming the north part of the parking lot into the pervious ground can be seen as progress toward increased resilience and climate adaptation (Fuss and O’Neill 2017).

### Jordan Cove Barrier Island: Highly Dynamic Barrier Island and Proactive Coastal Planning and Management Strategy

**Fig. 8:**
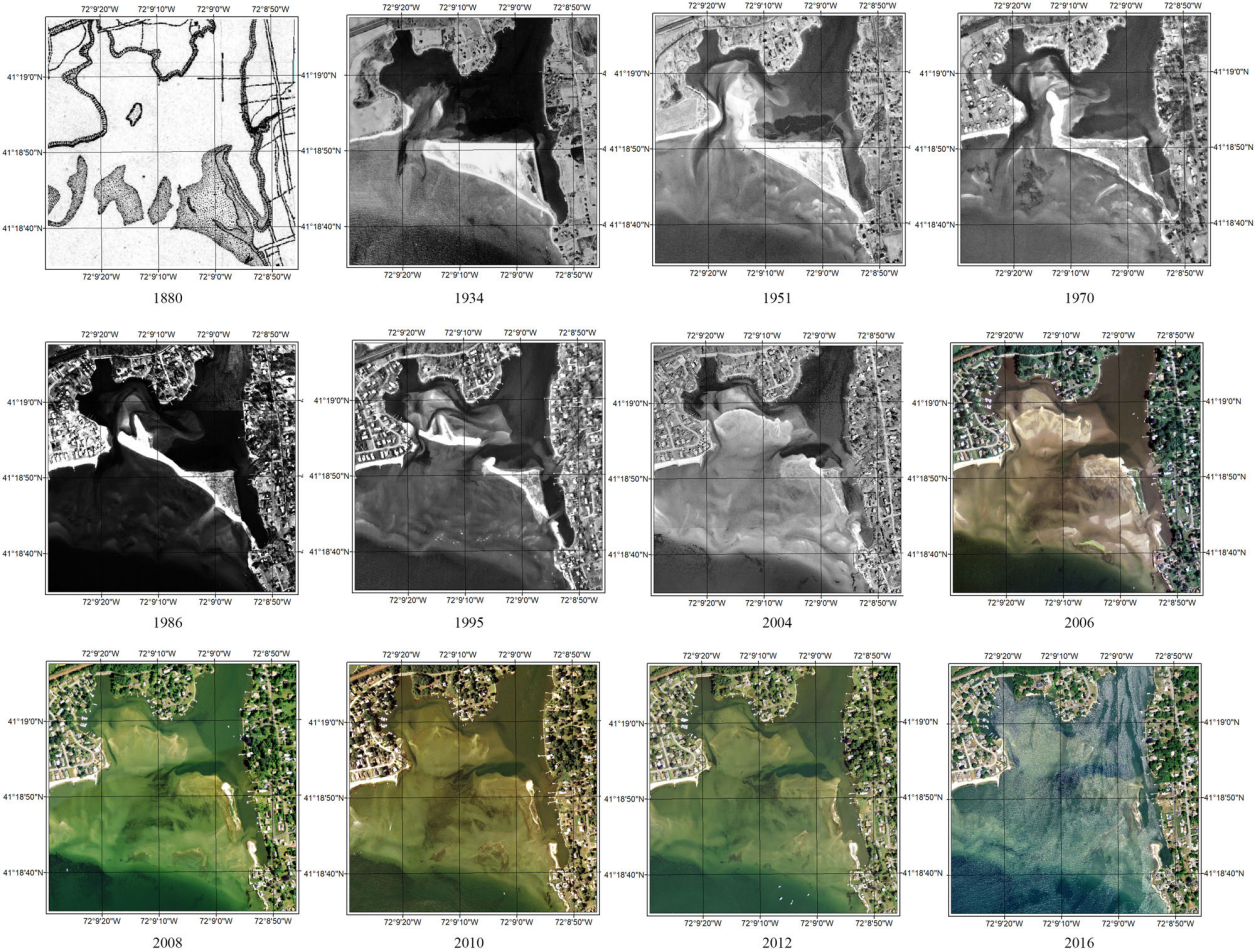
The time series of Jordan Cove barrier Island changes from 1880 to 2016

**Fig. 9:**
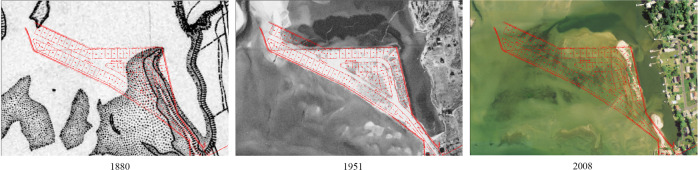
Overlay of 1948 plan with the map of 1880, aerial photos of 1951 and 2004 (from UConn CLEAR Connecticut’s Coast: Then and Now)

Jordan Cove barrier island in Waterford is an estuarine embayment with high dynamics along LIS. According to the historic map and aerial photographs from the 1880s, the shape of the barrier island constantly changed and ultimately disappeared at the beginning of 2000 due to coastal erosion and SLR (Fig. 8). Under the background of urban sprawl in the 1940s and 1950s, this island was seen as a “good” place for new development since it was in a relatively stable status. The local community proposed a subdivision plan in 1948 after land reclamation, but the city’s decision maker did not approve it then, which avoided property and life loss in the long run (Fig. 9). The present-day area is now shallow open water and tidal wetlands functioning as a unique environmental and resilience resource. It reveals the importance of considering long-term land-ocean change when making a decision and is an enlightening case for decision-makers.

### Coastal Land Geomorphic Change Analysis

**Fig. 10:**
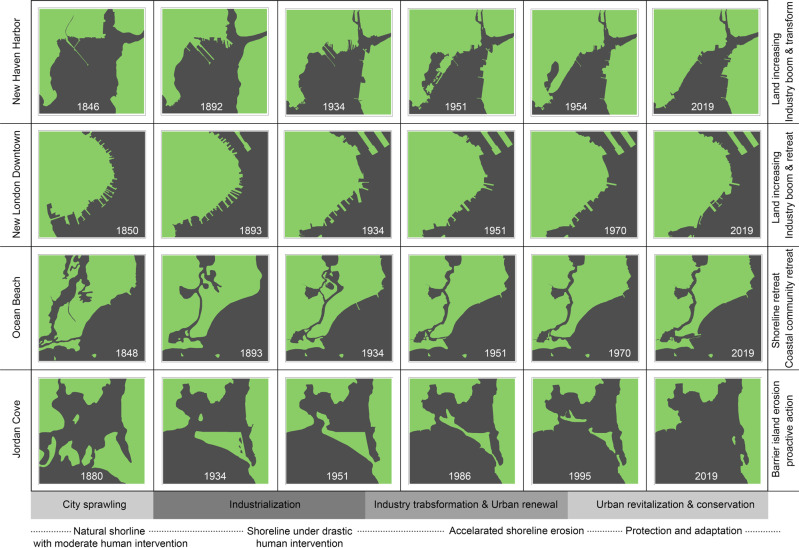
Four models of coastal land geomorphic changes and correlation with the social-economic changes

In the four types of coastal land physical change, New Haven harbor experienced dramatic land increase and waterward shoreline movement which created large tracts of artificially filled land for industrial and transportation uses. New London downtown had a highly industrialized shoreline in early times and maintained stability due to the deep-water harbor topography and conservation, but the port-related infrastructure and buildings, which once were densely distributed, gradually retreated landward to avoid the risk of extreme storms and coastal flooding. Ocean Beach shoreline maintained a landward trend due to erosion and SLR, although sand nourishment offset part of the loss; the community on Ocean Beach experienced wane and wax that represents an adaption process to respond to changing climate and public needs. Jordan Cove barrier island shows the extreme dynamics of barrier island and its unsuitability for development (Fig. 10).

### Coastal Land Area Changes and Trends Analysis

The four models of coastal land change and trend analysis show a similarity of fluctuating curves and overall declining trends in the long term. The rapid coastal development occurred in the mid-19th century and peaks in the mid-20th century which was a common process in most industrialized areas, especially in New England region. During this period, cities experienced intensified industrial development and urban sprawl resulting in a coastal land increase. In the latter half of 20th century, coastal land change slowed down and tended to stabilize under the background of industrial transformation and urban renewal, which represents a general global trend as well. With increasing rates of SLR and the profound influence of urban revitalization and conservation, the landward shoreline movement accelerated and coastal land shrank, accompanied by community retreat.

### Coastal Land use Change (CLUC) and Driver Analysis

The four typical CLUC cases demonstrate in New England that coastal land use is an outcome of the long-term interaction of coupled natural forces and human activities. In this process, humans continue to explore a balance between exploitation and adaptation, development and conservation. All four sites show the impacts of coastal erosion and land loss from SLR, with the most extreme case of Jordan Cove barrier island, in which significant land loss limited the potential for development. Overall, Ocean Beach’s size is decreasing but varies over time due to several episodes of beach nourishment; by relocating the community and utilizing the site as an open space, the vulnerability was reduced, and resiliency increased. Both New Haven harbor and New London downtown waterfront land use change are driven by industrial development and transformation as the cities’ economic pattern changes. However, as the focus shifts to public environmental consciousness and risk awareness, green infrastructure which introduce nature-based solutions is gradually being incorporated into these areas to increase resiliency. The retreat along the New London downtown waterfront properties and Ocean Beach community reflects the significant impacts of extreme weather events and adaptation strategies.

## Discussion

Change in social-ecological systems is neither continuous nor consistently chaotic, rather it is episodic (Franklin and MacMahon, 2000) with periods of slow accumulation of natural resources and physical structures, punctuated by sudden release and reorganization as a result of internal or external disturbances. From the CLUC analysis, rare events, such as hurricanes and large-scale infrastructure constructions, can unpredictably shape the structures of the coastal area and increase vulnerability, the long-term and slow variables dominate and enhance the overall trends of the change. Humans adapt well to changes in fast variables such as flooding, but are less successful in adapting to variables of intermediate rates, such as urban sprawl, and we are least successful in adapting to slow variables, such as climate change and the depletion of natural resources (Gunderson and Holling, 2002; Walker et al. 2006).

**Fig. 11:**
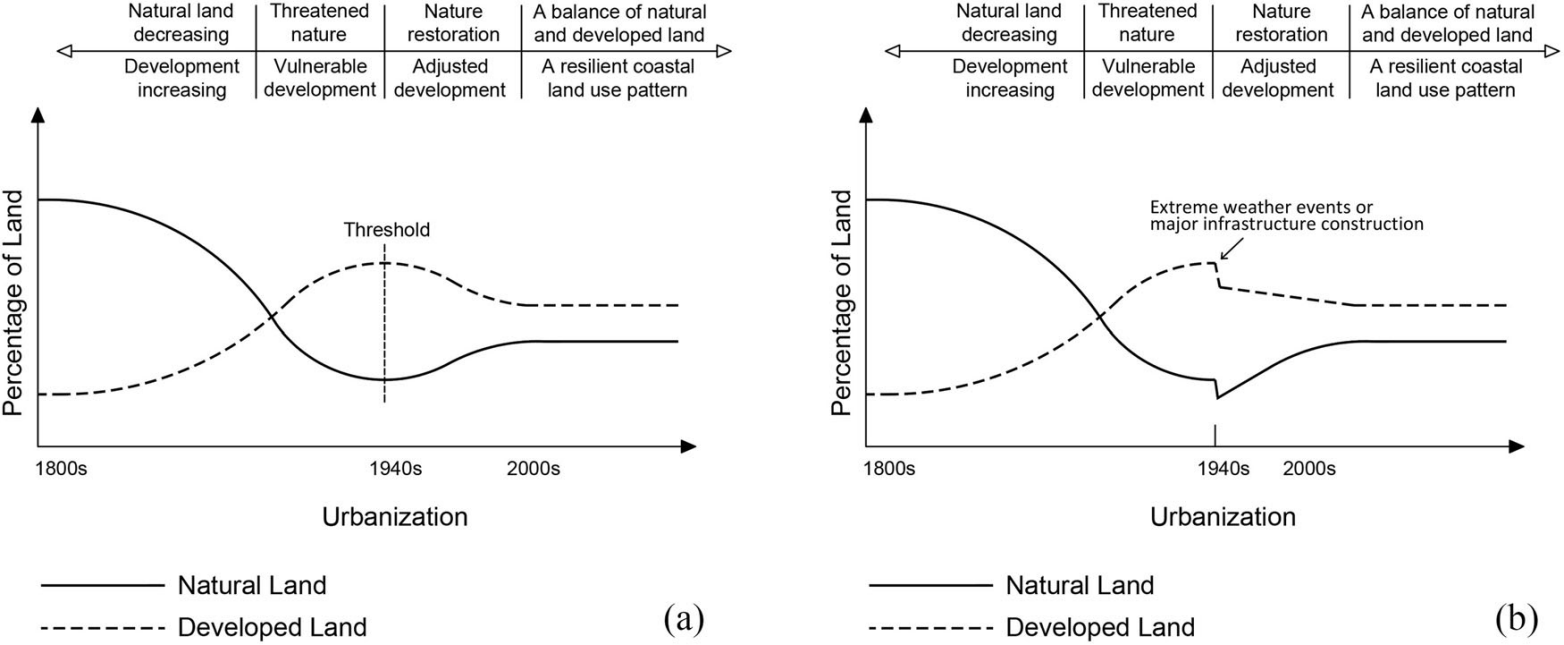
The coastal land use change (CLUC) model. **a** Model of land use changes under long-term drivers. **b** Land use changes under long-term drivers with short-term factors (or shocks)

The coastal land use change (CLUC) model shown in Fig. 11 demonstrates the ideal curves of typical coastal land use change patterns under coupled natural and human influences of long-term variation (population growth, urbanization, industrialization, shoreline erosion, and sea level rise, etc.) and short-term factors (hurricane, major infrastructure construction, etc.) in recent two centuries in Connecticut. The natural land kept losing due to urban sprawl and human activities along the shoreline until the threshold arrival that was caused by industrial transformation, institutional change, or extreme weather events. The trend after 2000s and the future may be a relatively stable status under the sustainable and resilient management strategy by balancing conservation and development proactively.

**Fig. 12:**
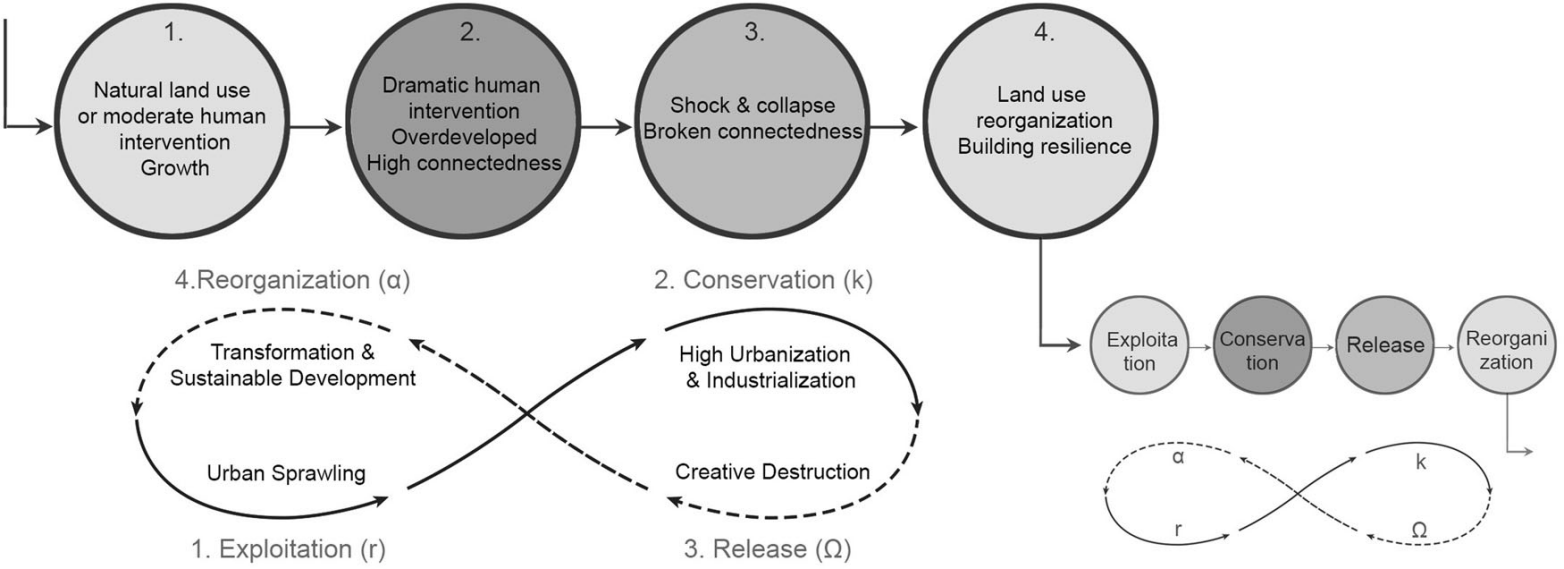
Adaptive cycle of coastal land use change and evolve. The adaptive cycle of CLUC demonstrates the process of building coastal resilience and seeking sustainable development. Phase 1–4 respectively represent exploitation (r), conservation (k), release (Ω), and reorganization (α). The solid lines represent the slow “front loop”, and the dashed lines are the fast “back loop”. In the “r” and “k” phase of front loop, the unregulated urban sprawling and over industrialization make the coastal area rigid and vulnerable, the “creative destruction” in “Ω” phase provides opportunities to break the high connectedness of controlling variables, the released resources can be reorganized in “α” phase to build a better land use configuration which is supposed to be more adaptive to the changed environment. The cycle will go on to keep the new development and policy adaptive to new situation and maintain sustainability

The destabilizing forces of disturbances, such as hurricanes and social-economic transformations, are important factors in maintaining diversity, resilience, and opportunities. For Ocean Beach, the 1938 New England hurricane was an opportunity to rethink land use and reinforce coastal resiliency. The industrial transformation is the determinant for New Haven harbor and New London downtown waterfront land use change, and an impetus to introduce green infrastructure and other resilience strategies. In the adaptive cycle, abrupt change can be creative destruction breaking the connectedness (or the rigidity) of internal controlling variables and processes (Fig. 12). Systems controlled through rigid variables and processes are limited in their resilience. Creative destructions free the resources, providing the opportunity to reorganize the combination of resources that is potentially better adapted to the environmental conditions and future trends (Gunderson and Holling, 2002). The opportunity brought by the release and reorganization phases may help rebuild resilience under long-term observation and understanding of the social-ecological system change. Ocean Beach is a case of successful adaptation to abrupt change and resource reconfiguration after destruction. As a matter of fact, the improved coastal land use is the consequence of rebuilding and reorganizing after disturbances. Reorganizing may lead to a better configuration of land use, resource utilization management, and institutional policies.

Stabilizing forces are also important in maintaining productivity and effectiveness, such as engineered protection strategies. However, policies and management that apply fixed rules for achieving stable states lead to the system increasingly losing resilience (Holling, 1995). The system may suddenly break down in the face of disturbances that previously could be absorbed and recovered from, such as engineered flood mitigation measures. The rigid systems eventually fail because they cannot take uncertainty into account and try to maintain an absolute stable status.

The previous coastal management emphasis by policymakers is on fast variables, short-term and partial goals. However, a profound understanding of slow variables, long-term impacts and changes compel people to focus on comprehensive goals and solutions with collective actions. Disaster response and hazard management in the United States have dramatically evolved. For most of the twentieth century, flood control policy in the U.S. has focused on controlling shorelines with structures such as surge barriers, floodwalls and levees through the United States Army Corps of Engineers (USACE) and other Federal agencies under the Flood Control Act of 1936. Following the 1938 hurricane, many of the USACE engineered projects were developed throughout New England (Morang, 2016); these engineered approaches rely on historical data, while floodplains and coastlines are dynamic landscapes that are continuously changing and evolving in a way that these structural solutions cannot accommodate. Also, these structural solutions often have negative environmental consequences over the long term and are especially detrimental for coastal natural resources migrating landward with sea level rise.

The resilient zone, such as wetlands and green open space, may become of critical importance for generating and maintaining resilience after disturbance and disruption (Smith et al. 2018; 2017). In contrast to conventional social-ecological management that aims at removing disturbance, resilient management focuses on building the ability of absorbing and recovering from a disturbance. The Ocean Beach community retreat and New London downtown waterfront retreat can be seen as a resilient management policy that is trying to maintain the coast as a resilient zone. New Haven harbor adopted the floodplain policy in 2010 that restricts land clearing activities and development of low-lying areas and encourages living shorelines, green infrastructure incorporated with pervious ground surface, to increase its resilience. Natural and nature-based features (NNBF), such as living shorelines, create and maintain natural habitats while providing resilience. The promotion and use of NNBF by federal entities such as the USACE, EPA (US Environmental Protection Agency), NOAA (National Oceanic and Atmospheric Administration) and USFWS (US Fish and Wildlife Service) as well as numerous non-governmental organizations and state/municipal governments is a step forward in planning and implementation efforts that recognize the detrimental aspects of structural solutions while promoting the resilience provided by natural habitats.

Therefore, coastal management policies must embrace uncertainty and unpredictability when formulating strategies that will stand the test of time in the face of our changing environment. Coastal management has to be flexible, adaptive, and experimental (Gunderson et al. 1995) to achieve the goal of sustainability, as well as long-term resilience.

## Conclusions

The four models of CLUC reveal how natural forces shape civilization and how human responses to natural hazards, which may help people understand the coastal dynamics and provide a base to make wiser decisions on land use. These models also demonstrate how human attempts to harness and transform nature using engineered, institutional and natural strategies, and how resilient management and planning can be incorporated to shape a sustainable coastal zone in the future. In this nature-human interaction process, it is crucial for decision maker and planner to understand the intrinsic dynamics of the past and the trends of the future, and to balance development and conservation to maintain the ability to absorb the forces of environmental uncertainty.

Urban planning and management strategies play a critical role in shaping coastal land use and managing coastal resources, and may guide a region to redevelop in a safer and more resilient direction after a change event. New London planning and zoning regulations seek to limit additional development in hazardous locations along the waterfront. Land acquisition of floodplain and open space preservation are encouraged as a municipal priority. Proactive decision-making in Jordan Cove and the resilience plan of New Haven and Ocean Beach represent resilient thinking in management when dealing with coastal hazards and the rapidly evolving coastal landscape.

In sum, facing a constantly changing shoreline and the challenges of climate change, coastal planning, management, and decision-making need to adaptively incorporate both long-term changes and the uncertainty of abrupt shocks. To achieve the goals of sustainable development, a resilient coastal land use and management strategies should be developed. A resilient management process must incorporate a cycle of learning, experimenting, and creating with the goal of developing new solutions that are able to deal with our ever-changing environment.
